# From Normal to Viral Body: Death Rituals During Ordinary and Extraordinary Covidian Times in Pakistan

**DOI:** 10.3389/fsoc.2020.619913

**Published:** 2021-02-16

**Authors:** Inayat Ali

**Affiliations:** Department of Social and Cultural Anthropology, University of Vienna, Vienna, Austria

**Keywords:** COVID-19, pandemic, rituals and ceremonies, death and dying, standard operating procedures (SOPs), liminiality, Sindh, Pakistan

## Abstract

Death is far from being simply a physiologic event; it is a complex phenomenon with sociocultural and politicoeconomic aspects. During extraordinary times such as the 2020 coronavirus pandemic, death becomes a contested site. I argue that the Pakistani government's dealings with the bodies of people who die from COVID-19 have shifted the meaning of a normal dead body to a *viral body* that poses particular challenges to cultures and people, including the government. This article is both autoethnographic and ethnographic. It concurrently draws on my observations and participation in death rituals in a Pakistani village in Sindh province as a member of that society, and on a recent experience that I faced after the death of a gentle lady of my acquaintance due to COVID-19. I also build on my previous long-term ethnographic research in Pakistan and my ongoing research on COVID-19 in that country. I discuss the death rituals and ceremonies performed during “ordinary” situations as background information; and the changes in these rituals that have resulted from the coronavirus pandemic. My data demonstrate significant differences between usual and customary death rituals and those performed during Covidian times by government mandate, which have severely and negatively affected people's mental health. I show the government's “symbolic ownership” of the viral body, in that the government can control how people deal with their *viral dead*.

## Introduction

Due to its multiple dimensions, death has been an interesting subject matter for many disciplines. Anthropologists, in particular, have long studied the rituals and ceremonies that surround death across cultures (Hertz, 2004). The body of work most relevant to this article is the study of the relationships between mortuary rites and epidemics that reveals how the state controls the final rituals, including burials. Sometimes, due to government policies and/or practical necessity, viral dead bodies are even never shown to their families (Robben, 2018). Barry (2018) describes how during the Great Influenza pandemic of 1918–1920 (aka the “Spanish Flu”), in multiple US cities, so many people died that funerals became impossible and bodies were simply left on front porches and in the streets to rot until a city wagon finally came to pick them up and bury them in mass graves. Lipton (2017) observed how death rituals became a “principal concern of the international response” during the 2014–2015 Ebola viral outbreak in Serra Leone. This domain raises pressing questions about “who owns death?”; these questions lead to inquiries about sovereignty and authority (Grant, 2011; Bernstein, 2019). These cases reveal whose power counts, “who presides over the funeral, who certifies the death, who serves as custodian…,” (Engelke, 2019) and who can show legitimacy to “own” the body.

The body confirmed or suspected to have died from COVID-19 is what I call a *viral body—*a term that can be extended to include bodies dead from other viruses as well. Dead viral bodies critically demonstrate the politics and poetics of death. The Pakistani government enacts its “symbolic authority” to either own the viral body and deny the family performance of the final rituals or to allow family members to observe these rituals according to strictly enforced guidelines, known as standard operating procedures (SOPs). To enact this authority, the government has employed various intricate maneuvers, which I will examine herein (Ali and Davis-Floyd, 2020). I argue that such government dealings have shifted the meaning of a human body from a “normal” to a viral body that, either alive or dead, may pose severe challenges to the living, and especially to the government.

Presenting a painful autoethnographic account of a gentle lady who passed away due to COVID-19, I compare death rituals and ceremonies performed during normal times and during Covidian times. I illustrate what constitutes an “ideal” death from a local perspective and demonstrate the government's SOPs for dealing with dead viral bodies, in order to reveal the differences caused by the government's responses to COVID-19.

## Methods and Materials

This article draws on autoethnographic and ethnographic research that I have conducted in Pakistan or from a distance. The data for discussing normal death come both from my personal experience and from previous ethnographic work that I conducted for my masters, M.Phil. and Ph.D. degrees (Ali, 2007, 2011, 2020c). This earlier ethnographic research significantly addressed the rituals and ceremonies related to death in Pakistan, especially at the village level. My autoethnographic accounts stem from my participation in the final rituals of my relatives and friends in Pakistan, whose deaths brought me deep grief. I also autoethnographically describe my emotions around the sudden death of a dear and gentle lady from COVID-19, and present ethnographic findings from a mobile phone conversation about the grief and mourning practices of people whose loved ones lost their lives due to COVID-19.

Moreover, for this article, I have conducted content and document analyses of news reports and government documents pertaining to COVID-19 and the government-recommended burial rituals and practices. The data on COVID-19 are part of my ongoing project on COVID-19 in Pakistan, approved by the National Bioethics Committee of Pakistan (reference No.4-87/NBC-471-COVID-19-09/20/).

## Death Rituals and Ceremonies During Ordinary Situations in Pakistan

This section draws primarily on my previously conducted ethnographic research in Pakistan (Ali, 2011, 2020c). In my country, the first thing that the family does when someone dies is to send messages to relatives, friends, and acquaintances. In the past, messengers carried this information and announcements were made via loudspeakers from an area mosque. Nowadays, both mobile phones and loudspeakers are used. Once the deceased person is buried (see below), participants discuss the deceased person's qualities, condole with the family, and offer supplications. The condolences often contain the following sentences: “It is God's will. Death is a reality. We all have to depart one day. If one thing will remain here, it is God.” In the following sections, I describe the characteristics of “ideal” death and ideal final farewells, primarily for Pakistan's Sindh province.

### Dying From Old Age

Death in Pakistan is defined as “natural” when someone dies during old age. Although a prolonged disease is considered the cleansing of your bad deeds prior to your departure, many people pray that they should die without any extended ailment. Most often, elderly people pray, “Ya Allah! bus halandi khay hala'i” [“Oh, God! Let the moving (breath) move without any interruption”]. I well remember the prayers to that effect that my grandparents chanted every morning and evening while rolling their prayer beads around their fingers.

### Dying at Home

Ideally, one must die in their own home and not at someone else's home nor in a hospital or *Pardais* (another country). People pray for this as well. I have heard many people say that they need to go back to their “forefathers' home” to die^1^. This cultural understanding has also been shown in various local television series and movies. Furthermore, I know people who, after living in other places for years within Pakistan or abroad, have returned to die in the villages where they were born and grew up. In Sindhi, such home villages are called *Abāi Ggoth*. Married women, however, go to their husband's and son's homes to die.

### Dying in the Presence of One's Entire Family

Older people regularly pray that when they die, their relatives should be around them. I remember that my grandmother used to say, “May God keep you around when I die so that you should participate in the funeral rites and be among those who carry my coffin to the graveyard and bury me.” It is considered a blessing to have close family members around and for them to perform the necessary rituals, or what Van Gennep (2019) would call rites of separation: ablution of the body, final make-up, dressing the body in a white shroud called *Kafan*, carrying it on a *charpoy*—a locally made bed of woven ropes normally used for sleeping—on their shoulders to the graveyard, participating in the *Namaz-e-Janaza* (funeral prayer), and burying the body. These are the ideal ways to say the final goodbye. After burial, all the charpoys in the house are placed upside-down on carpets laid on the floors throughout the house, especially in the largest hall or room of the house where all the women sit, mourn, and sleep for the first three consecutive days, in a state of what Turner would call “liminality”—the “betwixt and between” aspect of rites of transition (Turner, 1979). Male family members do the same at an *Otaq* (a guest house for male members). Sitting, mourning, and sleeping on the floor symbolize pain, sorrow, and grief. And turning the charpoys upside-down and sleeping “down” on the floor instead of “up” on one's charpoy index the ways in which the family's lives were turned upside-down by the death.

### The Performance of *Namaz-e-Janaza* (Islamic Funeral Prayer)

People also wish that when they die, their *Namaz-e-Janaza* will be performed, in which all family, friends, and relatives should participate. This is also related to the national belief system, as most people in Pakistan are Muslims. The number of participants reflects the deceased person's degree of community respect, and having many people offering prayers can be a source of Allah's forgiveness.

### Gendered Burial

In this highly patriarchal culture, only men are allowed to carry and/or accompany the charpoy to the forefathers' graveyard (*Ābāi Qabarastan)*. It is ideal that these men be close family members (depending on who died), such as father, brothers, husband, sons, and grandsons. They lower the deceased into the grave and put the first pieces of clay over the body. These male family members do not cover the entire grave with the clay; they just initiate the covering and then the other male participants finish the process. Meanwhile, the women of the family stay inside the house to mourn. They do visit the grave on the third day after the burial, and then again on the seventh day, and may visit the grave for the next seven consecutive Thursdays (Thursday is considered an auspicious day since it welcomes Friday, which is believed to be the holiest day in Islam).

### The Performance of the Final Rites

After death and burial, other rites commence that the family should perform. The first thing that occurs soon after the burial is the communal meal prepared for the attendees. In Sindh, there is a ritual called *Tijho*, in which relatives, friends, and acquaintances gather at the home of the bereaved family 3 days after the burial to bathe the grieving family and offer them washed clothes. Women put henna on each other's heads, especially on the heads of the women closest to the deceased person. Henna is a locally available and affordable dye; it symbolizes happiness and the end of sorrow. Its red color is sign of life that symbolically conveys the strong message to the bereaved family that life does not stop with someone's death; it continues. On the day of the death of a close family member, some women put dust or clay on their heads as a symbol of sadness and sorrow. Thus, applying henna 3 days later replaces that dust/clay and brings normality to that family. These rituals constitute what Van Gennep (2019) would call “rites of integration,” just as the preceding rituals I have described above constitute rites of separation and transition.

On this third day, in another ritual of integration, if the deceased was an adult male, a turban is put on the elder son and if the deceased is an adult woman, then a scarf/wimple is put on her elder daughter's head to denominate the *Waras* (heir). Moreover, on this day, the carpets are taken away from the floors, and all charpoys in the house are turned right side-up. These practices too are done to make things normal again. It is a very important part of the ritual that only the closest relatives, and not the immediate family members, arrange the materials for the baths, such as water, soaps, and towels. This arrangement shows the family's social standing and also the providers' close connections to the grieving family. Not only does the mourning family bathe, but also all the participants, especially those who have remained at that house for three consecutive days after the death.

Thereafter, on seven consecutive Thursday evenings, in ongoing rites of integration, it is necessary to arrange communal meals that at a minimum should contain seven types of food items. Although local people do not state any specific logic behind the number “seven,” in Islam, this number is considered linked to Allah, as it is believed that He created seven heavens, seven earths, and the seven days of the week, and completed the creation of humans in seven stages. Family and friends are expected to attend all these meals. Moreover, one final meal should be offered, called *Khairāt* (the English equivalent is something like “alms”). This meal is arranged at a large scale, in which all relatives and friends should participate; the economically poor are also invited as a means of alms-giving. *Khairāt* mostly entails the cooking of rice—sweet and spicy. It is believed that *Khairāt* brings forgiveness for the departed soul; thus, *Khairāt* constitutes the final ritual of integration. Special prayers are also arranged for this event, especially the chanting of the appropriate verses from the Holy Quran.

## Death and COVID-19: An Autoethnographic Account

It is 24 September 2020. I am writing this article on the same day when I have lost the dearest and gentlest lady of my acquaintance due to COVID-19. A day before her death, I was sent a photo of her sitting on a hospital bed with mild COVID-19 symptoms and food in front of her. The next morning, I see a picture of her grave. I am shocked and broken. Since at that moment I am sitting in the city center of Vienna, I cannot cry although I want to. With a heavy heart, I start drinking my tears.

Of course, this sweet lady's sudden death has also shocked her entire bereaved family. One day after she was tested COVID-19 positive, her family members who were in physical contact with her were recommended by doctors to go home, observe self-quarantine, and be tested for the virus. The next morning, the hospital staff call them with the news that the lady has died alone in the hospital—the most feared kind of death in Pakistan. Due to the government's SOPs, only a few family members, including her elder daughter, can reach her body there.

Moreover, their grief has been significantly expanded when they had to follow the government's SOPs around the final rituals, including the burial of her viral body, as well as to observe physical distancing and self-isolation. The final Covidian goodbye is extraordinary. While wearing personal protective equipment (PPE), her elder daughter has performed the *Ghusal* (a bath as per Islamic teaching to cleanse and purify the body)^2^ and *Kafan* (shrouding of the late person in white cotton sheets) at the hospital with the help of a few hospital staff. The *Ghusal* and *Kafan* otherwise could have been done by a religious lady with the help of some other women, including the sweet lady's daughters. And then the deceased sweet lady has been put in a coffin by the hospital staff while keeping her face slightly visible so that family members, relatives, and acquaintances can view her for the last time before her coffin will be brought to the graveyard by workers and a few male family members. Locally, this viewing is called Ā*khri Didār*. Since it is not allowed by the government, many relatives and friends cannot gather to participate in the *Namāz-e-Janāzā* (funeral prayer) and burial as they would have done in ordinary times. Even her second daughter cannot come to attend the final goodbye due to lockdown and travel bans. Except for her elder daughter wearing PPE, no other family can touch the late person, as the government has recommended, “Family and friends may view the body but should not be allowed to touch or kiss.” (Government of Pakistan, 2020).

Her death has significantly affected her elder daughter's mental and emotional health, who after many days passed still shares with a heavy heart, “I could not believe that our mother has died. I keep wanting to go back to her hospital room to find her, but I cannot, as my family will think I am gone mad. I still cry too much and remember our mother very much. I feel a mother is the only one who wants us to be truly happy. Despite all rights, she never asks us to serve her. In contrast, everyone else wants us, women, to serve. Every day is a new day in which you have to prove your worth.” Here this elder daughter speaks to the strongly patriarchal nature of Pakistani society, in which women serve men and feel heavily burdened by their multiple tasks. Her grief over the loss of her mother is compounded by the fact that her mother, despite her right as an elder to be served by the younger women of the house, was the only one who asked nothing of her, only gave.

## Death Rituals and Ceremonies During COVID-19

By 3 December 2020, Pakistan had reported COVID-19 infection in over 406,800 people, out of which over 8,200 have “officially” died (Johns Hopkins University, 2020). This may seem like a very small number of deaths out of so many cases; in other work (Inayat Ali, unpublished), I have shown how the Pakistani government may be fabricating these numbers in order to make it appear that they are doing an excellent job of coping with COVID-19. Pakistan reported its first infections in two men on 26 February 2020 (Ali and Salma, 2020). The government then issued a three-page document, *Burial and Safe Management of COVID-19 Dead Body*, which has affected the first five categories above related to death rituals, as I will describe below.

### Burial by Family Members or for Deaths at Home

I present these government guidelines for managing the *viral dead* one by one, listing the current governmental mandates and describing how these conflict with the usual sociocultural practices around death. My analysis does not constitute a critique of the government guidelines, as they are reasonable given the realities of COVID transmission (Rani, 2020). Some studies have documented the possibility of disease transmission from a corpse to other people who come into contact with it (Joob and Wiwanitkit, 2020). To avoid the transmission and provide a possible shield to people involved to deal with the dead bodies, a new safe design body bags have been introduced at the world level (Patel et al., 2020). Instead, my analysis intends to highlight the differences between practices during ordinary and Covidian times.

Family and friends may view the body but should not be allowed to touch or kiss and should wash hands thoroughly with soap and water frequently (Government of Pakistan, 2020).

During normal times, there is no restriction on touching the deceased person, although kissing is not encouraged. As soon as someone dies at home or in a hospital, as described above, the family moves the body to the biggest room in the house, where they sit on a carpet on the ground, start mourning, and frequently touch the feet of the deceased to show emotions and attachment. In some cases, the closest family members, e.g., sons, daughter, wife, and mother, may kiss the feet and the face. The female relatives, friends, and acquaintances who come by join them on the floor.

The burial rituals (burial gathering and prayers), if any, should have minimal possible numbers (only immediate family and relatives). All in attendance should observe standard precautions, i.e., social distancing of at least 2 m, facemasks, and frequent hand washes (Government of Pakistan, 2020).

As noted earlier, the *Namaz-e-Janaza* is a component of an ideal death. In ordinary times, there is no limit on the number of people who may participate in these rituals. High numbers are socioculturally encouraged, as they show the character, prestige, and position of the late person. Participants require neither physical distancing nor masks or washing of hands.

The family member preparing the body for burial should follow the standard precautions of wearing appropriate surgical/medical mask and/or gloves (Government of Pakistan, 2020).

In ordinary times, those who prepare the dead person for the final rituals, including burial, do not use any PPE. Nonetheless, they take a proper *Wuzu/Wudu* (Islamic ablution) prior to the preparation, which is an Islamic procedure to cleanse and purify specific body parts, such as washing the face, arms, and feet and cleansing the nostrils and mouth. Yet this cleansing is not re-performed after the burial; its purpose is not hygiene *per se* but rather to be clean and pure as they honor the dead.

Clothes worn by the person preparing the body should be immediately removed after procedure, washed with warm water at 60–90°C (140–194°F) and laundry detergent or a disposable apron/gown should be used (Government of Pakistan, 2020).

Usually, this is not the case. It is even culturally encouraged not to take one's clothes off for the next 3 days after preparing and burying the body to symbolize one's desire to hold onto the deceased and to demonstrate the pain and grief caused by the departure.

Immunosuppressed persons with underlying health conditions and adults > 60 years of age should not directly interact with the body (Government of Pakistan, 2020).

With no gender or age restrictions, ordinary times do not discourage anyone from interacting with the dead body. These are mostly the closest family members, and mainly women, who come into contact with the deceased person. They sob and ululate while surrounding the deceased.

Anyone handling the belongings of [the] deceased should wear gloves. The belongings should be disinfected with 70% ethanol. The household should be disinfected using 0.5% chlorine or 0.1% bleach solution (Government of Pakistan, 2020).

The clothes of the deceased or fabrics used like linen, towels, etc. should be washed in a machine using laundry detergent and warm water at 60–90°C (140–194°F) (Government of Pakistan, 2020).

This SOP highlights the urban/rural divide and the bias toward the urban that is common in Pakistan, as many rural people either have no washing machine or no sufficient electricity to run one (Zaidi, 1985; Ali and Ali, 2020). Thus, they cannot follow this SOP; instead, they wash the deceased's clothes with soap by hand, under a handpump, or at the bank of a small canal in normal water. Since simple soap kills the virus, it would have been much more appropriate and egalitarian for the government to take rural people into consideration in the guideline above. Regarding gloves, other than healthcare providers, during normal times, no one is obliged to wear them while preparing the deceased for the final rites. Customary sociocultural patterns do not require disinfection of the belongings nor the household. And, more interestingly, these patterns encourage the distribution of the deceased's possessions, such as clothes and shoes, among participants, or they are donated to economically needy persons as alms in the belief that this distribution would bring rewards to the deceased person. Such alms are of course not given for the viral dead.

According to media, in some places such as Karachi—the capital of Sindh province—specific graveyards have been designated for the burial of Covidian viral bodies (Raza, 2020). In April 2020, instead of family, government officials such as the Deputy Commissioner (DC) and the Station House Officer (SHO—the official in charge of a police station) attended the funerals of 10 people who died due to COVID-19. These officials and the graveyard's administration performed all funeral procedures, including Namaz-e-Janaza. The families never even saw their loved ones' bodies, indicating the full government ownership of these particular viral bodies.

The father of one young boy who died from COVID-19 told the media that only five family members were allowed to participate in Namaz-e-Janaza (Raza, 2020). The family's pain and sorrow were multiplied when the rescue officials showed reluctance to touch the coffin, which then was pulled from the ambulance with ropes (Raza, 2020). In such ways, customary grieving rituals are aborted, leaving people confused, upset, and at a loss for how to properly grieve. This discontent has resulted in the creative live streaming of funerals so that relatives, family members, and acquaintances can participate at a distance. But this does not work in rural areas, as not everyone owns a mobile phone, and often there is no electricity to charge phones nor sufficient bandwidth to establish a stable Internet connection.

The literature is still scant on death rituals during Covidian times in Pakistan, especially to explore what happens when the government really does take ownership of viral bodies and their families do not get to see them at all. Nonetheless, when there is no direct government ownership, then at least a “symbolic authority” of the government has emerged that enables it to recommend and enforce the necessary evidence-based protocols for burying COVID-19 infected dead viral bodies. Governments across the world, including Pakistan, are following and enforcing medically recommended guidelines for the treatment of viral bodies (Dijkhuizen et al., 2020; Raza, 2020).

## Conclusion

Death is far more than a physiologic event; it is a complex phenomenon that has sociocultural, economic, and political aspects. During extraordinary times, death becomes a contested site where these aspects become entangled, as the pandemic has revealed. Not only have these contestations affected the performance of final rites in Pakistan by relatives, but also they have impacted how and where those who have contracted the virus die. The pandemic's effects are severe in the country (Ali, 2020a,b; Ali and Davis-Floyd, 2020; Ali et al., 2020). In this article, I have demonstrated an ideal death in Pakistan and how, while the cultural ideals have remained the same, the practices that constitute an ideal death have had to change during this great pandemic when deaths are far from “ideal.” I have shown how a biological body becomes a “viral body” and triggers political negotiations around it. Considering this viral body as an extraordinary body that poses serious threats, the government has enacted its “symbolic ownership” of these viral bodies and has re-regulated the entire set of rituals and ceremonies normally performed after death. Given the six major characteristics of an ideal death in Pakistan, I have shown how government policies have affected five of them; these include: (1) dying at home; (2) dying in the presence of one's entire family; (3) the performance of *Namaz-e-Janaza* (Islamic funeral prayer); (4) male family members carrying the charpoy to the graveyard and burying it; and (5) the performance of the final rites. Effectively, the government owns these “viral bodies” in that it can control how they are treated. Government-imposed SOPs have affected people's mental health, in that their ability to mourn their dead in culturally normative rites of passage has been negatively altered. People fear contracting the virus, going into isolation, and dying without their near and dear ones around them, and their families fear the same. In Covidian times in Pakistan, as elsewhere, the families of the dead viral body must simultaneously face the grief of an unwanted departure and interrupted final rituals and rites that now cannot bring the closure for which they were culturally designed.

## Data Availability Statement

The datasets presented in this article are not readily available because: Since the data is confidential, it cannot be shared. Requests to access the datasets should be directed to Inayat Ali, inayat_qau@yahoo.com.

## Ethics Statement

The studies involving human participants were reviewed and approved by National Bioethics Committee of Pakistan (reference No. 4-87/NBC-471-COVID-19-09/20/). Written informed consent for participation was not required for this study in accordance with the national legislation and the institutional requirements.

## Author Contributions

IA designed the study, reviewed the literature, drafted, and revised the article.

## Conflict of Interest

The author declares that the research was conducted in the absence of any commercial or financial relationships that could be construed as a potential conflict of interest.

## Footnotes

^1^I use “forefathers” because it is the literal translation of local term *Ābāo Ajdād* and because Pakistani society, including Sindhi society, is patrilineal.

^2^Translation by the author.
